# Advances in clinical examination techniques for allergic rhinitis: a review from traditional testing to precision diagnosis

**DOI:** 10.3389/falgy.2026.1851667

**Published:** 2026-06-30

**Authors:** Xinyan Liu, Yongtai Wang, Hexiao Tian, Dongdong Zhu, Cuida Meng

**Affiliations:** 1 Department of Otolaryngology–Head and Neck Surgery, China–Japan Union Hospital of Jilin University, Changchun, China; 2 Jilin Provincial Key Laboratory of Diagnosis and Treatment of Upper Airway Allergic Diseases, Changchun, China; 3 Otolaryngology–Head and Neck Surgery Research Center, Changchun, China

**Keywords:** allergic rhinitis, artificial intelligence, biomarkers, examination techniques, precision diagnosis

## Abstract

Allergic rhinitis (AR) is an IgE-mediated, non-infectious inflammatory disorder of the nasal mucosa, and accurate diagnosis is important for individualized treatment. Over the past decade, diagnostic strategies for AR have evolved substantially from conventional methods, such as skin prick testing and serum-specific IgE testing, toward advanced approaches, including molecular allergen diagnostics, biomarker profiling, functional imaging, and artificial intelligence-assisted assessment. This review provides a systematic overview of these developments by summarizing the underlying principles, clinical utility, strengths, and limitations of each method. It also discusses the integration of multi-omics and digital health technologies, which have the potential to support truly precise and individualized diagnostics. By critically reviewing current strategies and future research directions, this article aims to help clinicians optimize patient management and promote further development in the field of AR diagnostics.

## 1 Introduction

Allergic rhinitis (AR) is a highly prevalent allergic disease that imposes a considerable burden on patients' quality of life and on healthcare systems. It is characterized by immunoglobulin E (IgE)-mediated inflammation of the nasal mucosa following exposure to inhaled allergens, leading to symptoms such as sneezing, nasal congestion, rhinorrhea, and itching (1). AR affects all age groups, and its incidence continues to rise worldwide, particularly among children and adolescents (2, 3). Other allergic diseases, including asthma, atopic dermatitis, and food allergy, frequently coexist with AR, reflecting complex interactions among genetic, environmental, and immunological factors (4, 5). Although understanding of AR pathogenesis has advanced considerably, accurate diagnosis and effective treatment remain challenging because of disease heterogeneity and clinical overlap with other respiratory disorders (6, 7).

The diagnosis of AR has traditionally been based on clinical history, physical examination, and confirmation of sensitization to specific allergens using skin prick testing (SPT) or serum-specific IgE (sIgE) assays (8, 9). However, these standard procedures have limitations, including false-positive results caused by cross-reactivity and inability to detect local allergic responses in patients without systemic sensitization (10, 11). The concept of local allergic rhinitis (LAR), characterized by nasal symptoms and local IgE synthesis despite negative systemic allergy testing, has emerged as an important phenotype and has stimulated development of more specific diagnostic methods, including nasal allergen provocation testing (NAPT) and nasal sIgE measurement (10, 12). Differentiating LAR from classical AR and non-allergic rhinitis (NAR) has important diagnostic, therapeutic, and pathophysiological implications (13, 14).

The pathophysiology of AR involves a complex sequence of immunological events triggered by allergen exposure. These events include activation of Th2 cells, type 2 innate lymphoid cells (ILC2s), mast cells, and eosinophils, with subsequent release of cytokines such as interleukin (IL)-4, IL-5, and IL-13 (1, 15). This type 2 inflammatory response promotes IgE synthesis, mucosal edema, and nasal mucosal hyperresponsiveness. Recent evidence also implicates epithelial barrier dysfunction, neuroimmune interactions, and inflammatory pathways such as NF-kappaB and mitogen-activated protein kinase (MAPK) signaling in the initiation and persistence of allergic inflammation (7, 16, 17). In addition, genetic and epigenetic factors contribute to individual susceptibility and disease heterogeneity, and recent studies have identified key genes and regulatory RNAs involved in AR pathogenesis (18–20).

Environmental factors such as airborne pollen, house dust mites, and air pollution contribute to AR symptoms, while seasonal and regional differences influence sensitization patterns and disease prevalence (21, 22). The increasing burden of AR and its comorbidities, including asthma and chronic rhinosinusitis (CRS) with nasal polyps (CRSwNP), highlights the need for integrated approaches to allergic airway disease management (6, 23, 24).

Current treatment options for AR primarily aim to control symptoms and include antihistamines, intranasal corticosteroids, leukotriene receptor antagonists, and allergen immunotherapy (AIT) (25, 26). Among these, AIT is the only established disease-modifying therapy capable of inducing long-term remission and reducing the risk of asthma development (26, 27). Nevertheless, challenges remain, including patient adherence, appropriate patient selection, and optimization of treatment protocols (28, 29). Emerging therapeutic approaches, including biologics targeting key cytokines and immune cell pathways, as well as natural compounds such as saponins and postbiotics including ectoine, are being investigated for their potential to modulate allergic inflammation with favorable safety profiles (30–32).

Digital health technologies, including mobile health (mHealth) applications and telemedicine, have improved patient monitoring, symptom tracking, and individualized AR management, thereby enhancing adherence and clinical outcomes (28, 33, 34). In parallel, artificial intelligence (AI) and machine learning (ML) tools are increasingly being used to analyze complex clinical and molecular datasets, refine phenotyping, predict disease progression, and optimize treatment strategies (35, 36).

**Figure 1:**
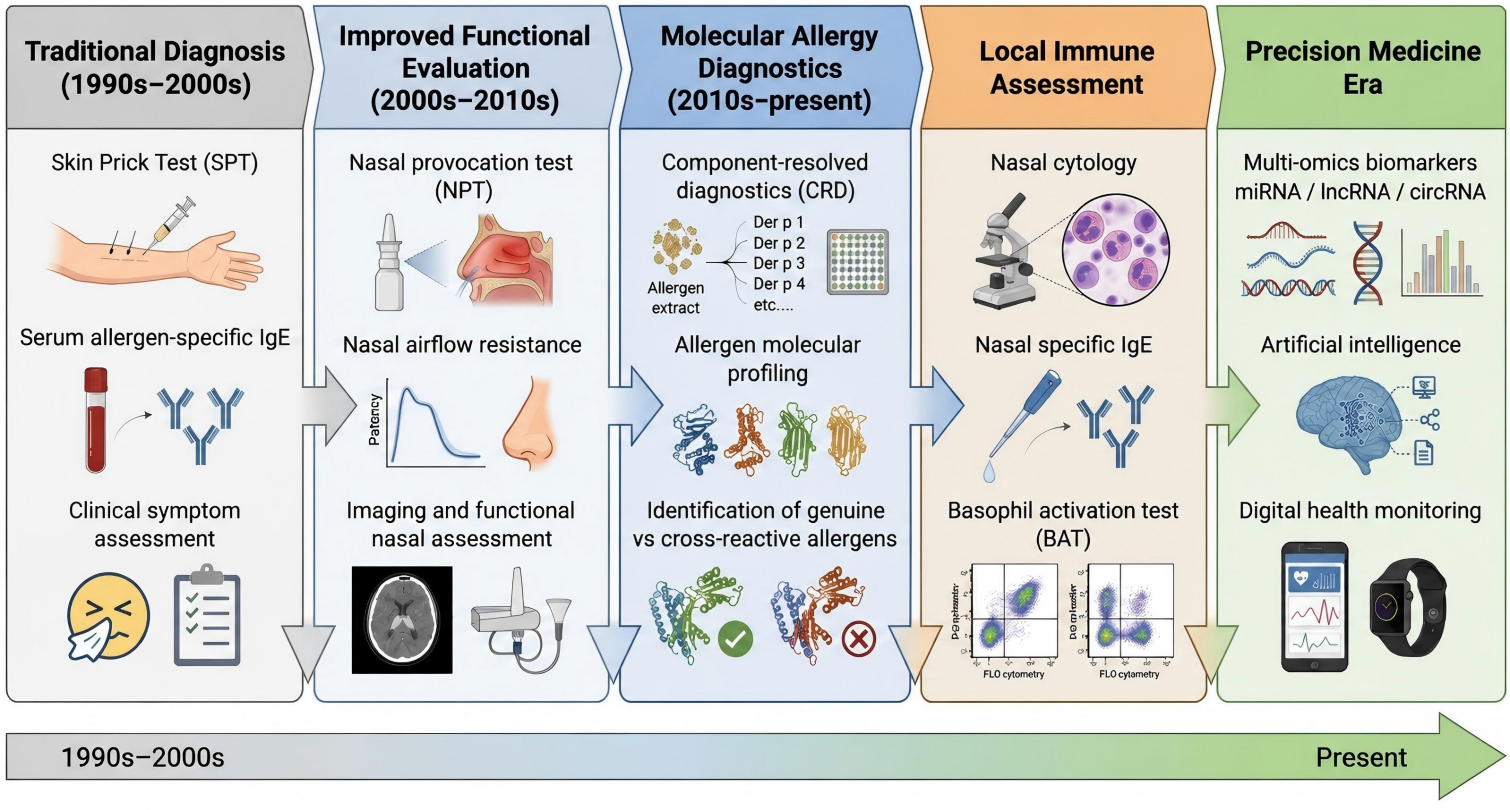
Evolution of diagnostic strategies for allergic rhinitis.

Over recent decades, diagnostic strategies for AR have evolved from conventional clinical evaluation and systemic IgE testing toward more precise approaches incorporating molecular evaluation, assessment of local immune responses, and digital health technologies. These advances have improved identification of disease phenotypes and endotypes, thereby facilitating personalized management strategies (Figure 1).

Overall, AR diagnosis and treatment are moving from symptom-centered approaches toward integrated precision medicine models that combine molecular diagnostics, patient-centered care, and emerging therapeutic opportunities. Development of effective individualized therapies requires a comprehensive understanding of molecular pathogenesis, environmental influences, and patient-specific factors. This review systematically summarizes recent advances in clinical examination techniques, molecular understanding, and diagnostic concepts in AR, providing a practical resource for clinicians and researchers seeking to improve diagnosis and management of this prevalent and complex disorder.

AR is primarily characterized by a type 2 (Th2) immune response involving cytokines such as IL-4, IL-5, and IL-13, which promote IgE production, eosinophilic inflammation, and mucosal hyperreactivity. These mechanisms underpin most diagnostic approaches discussed in this review. Subsequent sections will therefore focus on how different diagnostic technologies capture or reflect these shared underlying mechanisms.

Diagnostic approaches for AR have evolved from traditional clinical evaluation and systemic allergy testing toward increasingly precise and multidimensional strategies. Early methods primarily relied on SPT and sIgE detection. Subsequent developments incorporated functional nasal assessments and molecular allergen diagnostics, including component-resolved diagnostics. More recently, evaluation of local nasal immune responses, such as nasal cytology and nasal IgE detection, has improved identification of disease endotypes such as LAR. Emerging technologies, including multi-omics biomarkers, digital health tools, and artificial intelligence, are expected to further advance precision diagnosis and individualized management of AR.

## 2 Traditional diagnostic approaches

These approaches remain the foundation of AR diagnosis and are widely used in routine clinical practice.

### 2.1 Skin prick testing: standardization and quality control

A first-line diagnostic procedure for evaluating IgE-associated allergic diseases is skin prick testing (SPT) because it is a quick, inexpensive, and sensitive test. However, SPT results can be influenced by several factors, including the technique of the operator, quality of the allergen extract, skin responsiveness of the patient, and recent antihistamine use, which may suppress skin responses and produce false-negative results. These considerations underscore the need for rigorous standardization and quality control processes to increase the reproducibility of SPT results and comparability across different clinical settings.

#### 2.1.1 Standardization of allergen extracts and controls

Recent advances have focused on the standard operating procedures in allergen extract preparation, application methods, and standards of interpretation. Standardized allergen extracts of well-characterized, defined protein content, and defined allergenic potency are critical in minimizing variability in the results of SPT. To illustrate this point, it is shown that the stabilizing preparations containing glycerol with amino acids, such as arginine and glutamic acid, have the ability to stabilize the allergenic activity of the mold extracts over extended periods to ensure that the test is always performed (37). Similarly, the development of skin prick test solutions to occupational allergens in community pharmacies has been simplified to maintain equal protein integrity with commercial extracts, and the quality of extracts is critically important (38). In addition to the standardization of the allergen, positive and negative controls are required to ensure quality in SPT. Histamine is used as the positive control to verify the skin reactivity and saline or glycerol solutions are used as negative controls to remove the nonspecific skin reactions. Wheal size is typically interpreted to be positive to the allergen, as the size of the wheal is greater than 3 mm, or greater than that of the negative control by a defined margin, is considered a positive result (39). This makes the SPT interpretation more objective and facilitate laboratory comparability.

#### 2.1.2 Operator training and patient preparation

Operator training and standardized protocols are also important in ensuring that SPT is accurate. The effect of the lancet pressure, angle and duration of skin contact on the size of the wheal and sensitivity of the test may have a significant effect. Clinical guidelines therefore recommend comprehensive training programs and frequent competency assessments of staff who perform SPT in order to promote procedural uniformity (40). Patient preparation is also important; patients should stop taking antihistamines and other medications that interfere with the test before testing to prevent false-negative results (41).

#### 2.1.3 Complementary diagnostic approaches and clinical applications

Intradermal testing may supplement SPT with some allergens, particularly drugs and insect venoms, to increase diagnostic sensitivity. However, intradermal tests are more likely to cause systemic allergic reactions and require careful risk-benefit assessment and clinical observation (40). In this respect, intradermal testing should be applied in those situations when the SPT findings are inconclusive or negative but the clinical history is good.Recent diagnostic additions to SPT include measurement of specific IgE in serum and nasal secretions, and NAPT. Though serum and nasal IgE test can objectively identify the extent of sensitization, they might not be able to identify all local mucosal allergy. The reference standard for confirming the existence of nasal allergic reactions is called NAPT, which is the method that replicates the natural exposure to an allergen and quantifies clinical reactivity but is more time-consuming and resource-intensive (42). More recent advances,e.g., quantum dot immunofluorescence to measure IgE in nasal secretions in a short time, offer some promising non-invasive alternatives with high diagnostic concordance to SPT (43).SPT will remain fundamental in children to early identify allergic sensitization in order to make management and immunotherapy decisions. It has been revealed that age- and sex-specific differences in the patterns of allergen sensitization do exist, and the most common sensitizers in children with respiratory allergies are house dust mites (44). Normalization of SPT among children is of particular importance due to the contributions of allergic illnesses to the quality of life and comorbidities such as asthma and attention-deficit/hyperactivity disorder (45).

### 2.2 Serum-specific IgE testing: technical innovations and clinical interpretation

Automated systems such as ImmunoCAP and ImmunoCAP ISAC have enabled the detection of serum-specific immunoglobulin E (sIgE) more accurately and reproducibly. The technologies have significantly improved the standardization and throughput of sIgE testing, enabling the measurement of allergen sensitization with better reliability and quantitative values. For example, ImmunoCAP provides quantitative information in kilo units of allergen-specific IgE per liter (kUA/L), which can be used to determine the degree of sensitization to particular allergens by clinicians.This quantitative capability may support clinical decision-making since it enables the stratification of the patients based on the degrees of sensitization and could be applied to forecast the extent of the allergic reactions. Several studies have suggested that specific sIgE cutoff levels like 17.0 kUA/L with house dust mite (HDM) allergens can be predictive of positive nasal provocation test results with high specificity and positive predictive value and that quantitative sIgE measurements can be used clinically to diagnose and manage AR (8).

However, interpretation of sIgE results should be done with caution when it comes to clinical history and symptomatology. A positive sIgE result does not necessarily indicate clinical allergy because asymptomatic sensitizations are common. Asymptomatic sensitization refers to the presence of high sIgE without any allergic symptoms. Epidemiological evidence indicates that sIgE antibodies are more frequently present in patients with intermittent or persistent AR than in healthy controls, even when the skin prick test results are negative or weakly positive, and it is necessary to take sIgE positivity into account in the context of the clinical picture (46). Besides, sensitivity and specificity of sIgE test in the diagnosis of AR have been compared to other diagnostic methods, such as nasal smear eosinophilia with sIgE demonstrating a higher sensitivity and negative predictive value making it a useful diagnostic test when properly used (47).

A major limitation of traditional sIgE assays is that they employ allergen extracts that are complex mixtures of different protein components. This approach does not discriminate sensitization to individual allergenic proteins in an extract, thereby limiting the diagnostic accuracy. The absence of the possibility to identify the precise components of the protein that is sensitized is an impediment in the path to more precise allergy diagnostics. For example, allergic patients to different molecular components of the HDM allergens may have different clinical phenotypes and non-uniform responses to immunotherapy that cannot be identified using conventional extract-based sIgE methods (8). This has seen the development of component-resolved diagnostics (CRD), such as ImmunoCAP ISAC, which employs microarray technology to detect sIgE to multitudes of purified allergen components simultaneously. CRD has been reported to improve diagnostic specificity in selected patient populations, particularly in distinguishing cross-reactivity; however, its impact on overall diagnostic accuracy in routine clinical practice remains variable (48). Several studies have demonstrated improved component-level resolution, although consistent improvements in clinical outcomes have not been clearly established (49–51).

To ensure the reliability of sIgE in clinical practice, the stability and reproducibility of sIgE measurements has been extensively tested. It has been demonstrated that sIgE antibodies are not sensitive to various preanalytical conditions, including centrifugation delay, refrigerated storage, but long-term storage and repeated freeze-thaw cycles can lead to variation (52). This strength promotes popular use of sIgE in clinical practice.

Importantly, although both SPT and serum-specific IgE (sIgE) testing are widely used as first-line diagnostic tools, their diagnostic performance varies depending on clinical context and population. Reported sensitivities for SPT in AR typically range from 70% to 90%, with specificities between 80% and 95%, whereas sIgE assays demonstrate comparable or slightly higher sensitivity but may exhibit reduced specificity due to asymptomatic sensitization and cross-reactivity (46). In real-world clinical settings, discrepancies between SPT and sIgE results are not uncommon, particularly in polysensitized individuals or in cases with low-level sensitization. Moreover, both methods may fail to identify LAR, highlighting a critical diagnostic gap. These limitations emphasize that neither test should be interpreted in isolation, and their diagnostic value is significantly enhanced when combined with clinical history and, when necessary, nasal provocation testing. From a practical perspective, SPT remains more cost-effective and accessible, making it suitable for large-scale screening, whereas sIgE provides quantitative and standardized results that are less operator-dependent but at higher cost. Therefore, the choice between these methods should balance diagnostic accuracy, resource availability, and clinical necessity.

Collectively, advances in sIgE testing have improved standardization and quantitative assessment of allergen sensitization.

## 3 Molecular diagnostic approaches

To overcome the limitations of conventional methods, molecular diagnostic techniques have been developed to improve allergen specificity and support individualized management of IgE-mediated allergic diseases, including AR.

### 3.1 Component-Resolved diagnostics: recombinant allergens, principles, and advantages

Molecular allergology has redefined allergy diagnostics in molecular allergology by enabling identification of some allergenic proteins that drive sensitization is possible as opposed to using crude allergen extracts. Recombinant allergen diagnostics Recombinant allergen diagnostics use gene-engineered expression of purified allergenic proteins, recombinant or purified natural components, to detect allergen-specific IgE (sIgE) antibodies with high specificity. This technique, known as CRD, allows the molecular profiling of the sensitization of a patient with specific protein components that lead to an allergic response as compared to the use of whole extracts, which is a mixture of the allergenic and non-allergenic components (53).

A major advantage of CRD is that it is able to distinguish between primary sensitization and cross-reactive sensitization by structurally related proteins in various allergen sources. The pathogenesis-related protein Bet v 1, the major allergen in birch pollen, may cross-react with homologous proteins in food such as apple and peach to produce birch-apple-peach syndrome. The capacity to detect IgE antibodies selectively directed against Bet v 1 facilitates the clinician to identify true sensitization and cross-reactivity that is critical to diagnose and treat the disease, dietary counseling, and immunotherapy selection (53). Similarly, the important allergens of the major house dust mite, Der p 1 and Der p 2 have been fully characterized as the significant components that mediate respiratory allergy, and recombinant versions of these proteins have enhanced diagnostic sensitivity and informed allergen-specific immunotherapy (AIT) (54).

Recombinant allergens can also be used to identify the pan-allergens, which are extensively expressed proteins in different species and may cross-react, such as profilins and tropomyosins. Recombinant profilins of various pollen sources (Amb a 8, Art v 4, Bet v 2, Phl p 12) have been generated and validated and have been used to identify patterns of sensitization to aid in distinguishing between clinically important sensitizations and also to assist in differentiating between cross-reactivity (55). Likewise, recombinant tropomyosin allergens of crustaceans, such as Macrobrachium nipponense, are observed to retain an equivalent allergenicity to their natural counterparts, and have been exploited to the diagnosis of food allergies and in the potential treatment of immunotherapy (56).

Several limitations of extract-based diagnostics, such as variability in the allergen content, cross-reactive carbohydrate determinants (CCDs), which can result in false-positive IgE outcomes, can be overcome using recombinant allergens. To minimize the possibility of CCD interference, recombinant proteins are typically glycosylated in the non-glycosylated form or reduced CCD epitopes. Nevertheless, it has been established that even immobilized recombinant allergens on cellulose-based assay matrices can contain CCD-associated false positives that can be reduced by soluble CCD inhibitors or other assay media (57). These findings highlight the role of assay design and quality control in CRD.

Recombinant allergen-based diagnostic methods may improve sensitivity and specificity. For example, CytoBas that directly stains IgE on basophils using flow cytometry with fluorescently labeled recombinant allergen tetramers and enables the simultaneous and rapid detection of sensitization to single allergen components such as Lol p 1 and Api m 1. This technique has a high positive predictive value and enables simultaneous assessment of multiple allergen components within a single assay, representing a promising advancement in CRD (58). Similarly, IgE profiling global, a personalized diagnosis and personalized immunotherapy actions are supported by the use of microarray technologies on a panel of recombinant allergens (59).

Recombinant allergens must be produced with careful attention to protein folding and post-translational changes in order to preserve native conformations and IgE-binding epitopes. In order to obtain high-quality preparation of recombinant allergens that can be used as a diagnostic and therapeutic agent, quality control measures have been put in place which include chromatographic separation and IgE reactivity measures such as the case with Der f 2 allergen (60). The expression of recombinant allergens in expression systems such as Escherichia coli and Pichia pastoris has demonstrated similar IgE reactivity and yield and stability improvements with the addition of fusion tags without any change in antigenicity as demonstrated in the production of the dog allergen Can f 6 (61).

CRD can also be used to identify new allergenic components in multiple allergen sources and this widens the scope of known allergens and improves diagnostic coverage. An example of this is that recombinant protein technology has been utilized to characterize new allergens, such as Amb a 11, Tyr p 31 and Bro p 3 ragweed pollen, storage mites and Broussonetia papyrifera pollen respectively, and their clinical and diagnostic potential has been uncovered (62–64). These findings further expand current knowledge of allergen diversity and support the continued development of recombinant allergen-based diagnostic approaches of allergies.

In summary, recombinant allergen-based CRD represents an important advancement beyond conventional extract-based testing by providing a more precise and molecularly defined diagnostic approach.

### 3.2 Multiplex platforms: clinical applications of microarray technology

One notable advance in clinical diagnosis of allergic diseases, including AR, is the multiplex microarray chip technology including ImmunoCAP ISAC and ALEX. These microarray chips can be used to identify certain IgE antibodies to hundreds of allergen components using small serum volumes, which is particularly useful with pediatric samples or with patients who have only limited serum samples. This high-throughput capability is especially applicable in patients who are polysensitized, have a complicated clinical manifestation or incongruent results of traditional diagnostic testing, such as SPT or singleplex specific IgE tests. It is demonstrated that multiplex microarrays can be employed to map sensitization patterns to a large variety of inhalant allergens, including HDM components (Der p 1, Der p 2, Der p 10, Der p 23), pollens and animal dander, with favorable analytical performance (65). Recent sensitization patterns resolution capability allows clinicians to distinguish between genuine sensitization and cross-reactivity, which are crucial to correct diagnosis and personal treatment.

Among the most significant clinical uses of the multiplex microarray technology is the possibility to establish some sensitization patterns that could guide the pathophysiology and shape therapeutic decisions. By way of example, both Der p 1 and Der p 2 are commonly linked to classical HDM allergy but isolated sensitization to Der p 10, a tropomyosin could be an indication of cross-reactivity with other invertebrate allergenes, such as shellfish and not necessarily mite allergy (66). Such molecular-level discrimination may improve diagnostic precision and enables specific AIT depending on the pertinent allergen components. It was shown in the real world that the ImmunoCAP ISAC 112 microarray is capable of altering the immunotherapy prescription of more than half of polysensitized patients, which may influence clinical decision-making (67, 68). Moreover, the identification of the main allergens, such as Der p 23, associated with the asthma pathogenesis and the severity of AR, also suggests a potential role for CRD in risk stratification and prognostic assessment (69).

Despite these advantages, multiplex microarray technology to clinical practice has several challenges. One of the largest limitations is cost since such assays are still comparatively expensive to use relative to the traditional diagnostic methods and this could restrict their application particularly in resource-constrained environments. In addition to that, interpretation of multiplex results should be done with certain expertise since sensitization profiles are complicated and homologous elements of allergens can be cross reactive. Clinicians should use both molecular information and clinical history together with other diagnostic outcomes to avoid misinterpretation. Standardization and validation of assay platforms in a large number of populations is also necessary so as to ensure reproducibility and reliability. But the present developments in assay design, bioinformatics to analyze data and growing clinical experience are gradually overcoming these challenges.

Despite its high analytical resolution, CRD does not necessarily translate into superior clinical utility in all scenarios. While CRD improves molecular-level specificity and can differentiate genuine sensitization from cross-reactivity, its incremental diagnostic value over conventional SPT and sIgE testing in routine AR cases remains debated. In particular, the risk of overdiagnosis should be considered, as detection of IgE against minor or clinically irrelevant allergen components may not correlate with symptomatic disease. This may lead to unnecessary dietary restrictions or inappropriate immunotherapy selection. Furthermore, CRD assays are associated with higher costs and require specialized interpretation, which may limit their widespread implementation in primary care or resource-limited settings. Current evidence suggests that CRD is most beneficial in complex cases, such as polysensitization, unexplained symptoms, or prior to allergen immunotherapy decision-making, rather than as a universal first-line diagnostic tool (67).

In summary, the multiplex microarray chip technology can be an effective approach to a comprehensive assessment of the allergen sensitization at the molecular level, which could be used to diagnose AR and treat it individually. It is particularly sensitive to various components of allergen in low serum volumes, which is especially relevant to complicated cases of polysensitization or vague clinical presentation. The technology enhances the quality of the diagnosis and informs specific immunotherapy by clarifying some patterns of sensitization and differentiation in classical and cross-reactive responses. It is limited to cost and interpretative complexity but with constant advancements, multiplex microarrays will be able to be used more frequently in the practice of allergy diagnostics and with the potential to support more individualized diagnostic and therapeutic strategies (65, 67, 69, 70).

Given the increasing diversity of diagnostic approaches, a direct comparison of their analytical performance, clinical strengths, and practical limitations is important for guiding appropriate test selection in different clinical scenarios. Table 1 summarizes the key characteristics of commonly used diagnostic methods for AR. Data are derived from representative studies in the literature and may vary depending on population and methodology. In real-world practice, diagnostic performance is often lower than reported in controlled studies due to variations in patient characteristics, allergen exposure, and operator experience.

**Table 1:** Comparison of major diagnostic methods for AR (61, 65–68, 70).

Method	Sensitivity	Specificity	Advantages	Limitations	Clinical Role
Skin Prick Test (SPT)	High (∼80%–90%)	Moderate	Rapid, inexpensive, widely available	Operator-dependent; affected by medications	First-line screening
Serum specific IgE (sIgE)	Moderate–High	High	Quantitative; standardized	May detect asymptomatic sensitization	Confirmation of sensitization
Component-Resolved Diagnostics (CRD)	High	High	Molecular precision; distinguishes cross-reactivity	High cost; complex interpretation	Polysensitized or complex cases
Nasal Allergen Provocation Test (NAPT)	Very high	Very high	Gold standard for LAR	Time-consuming; limited availability	Confirmatory testing
Nasal Cytology	Moderate	Moderate	Non-invasive; reflects inflammation	Observer variability	Inflammatory phenotyping
FeNO/nNO	Variable	Variable	Non-invasive; real-time	Limited specificity	Airway inflammation monitoring
Imaging (CT, Endoscopy)	Moderate	Moderate	Structural and functional assessment	Cost; radiation (CT)	Adjunct evaluation
AI-based models	Emerging	Emerging	Integrates multi-source data	Requires validation; potential bias	Future precision diagnostics

As shown in Table 1, each diagnostic modality presents distinct trade-offs among accuracy, feasibility, and clinical relevance. These differences underscore the need for a stepwise and integrated diagnostic strategy rather than reliance on a single test. Although NAPT suggests high diagnostic accuracy, its limited availability restricts routine use, whereas SPT and sIgE remain primary tools in most clinical settings because of accessibility. However, molecular sensitization does not always correspond to clinical symptoms, and functional assessment may therefore be required.

## 4 Functional and biomarker-based diagnostics

Beyond allergen identification, functional and biomarker-based approaches assess clinical relevance, inflammatory activity, and local immune responses.

### 4.1 Local IgE/nasal biomarkers

#### 4.1.1 Quantitative and automated analysis of nasal secretion cytology

Eosinophil counts in nasal smears have been a cornerstone of the adjunctive diagnosis and inflammatory categorization of AR, and have been shown to provide useful data regarding the underlying immunopathology. However, the traditional manual counting methods are subjective and rely on the experience and ability of the operator and as such is likely to be variable and lack reproducibility between laboratories and clinicians. To address these weaknesses, new cell centrifugation smear techniques have been developed that better prepare the sample by centrifuging the cells to a more homogeneous sample, which improves the quality and consistency of cytological slides. To complement these developments, automated cell image analysis systems with advanced algorithms and ML have been developed to detect and measure eosinophils and other inflammatory cells objectively. Such automated systems can substantially reduce the human error and inter-observer variability, and are able to offer more reliable and standardized assessments. In addition, concomitant identification and measurement of other inflammatory cells in nasal secretions, including neutrophils and mast cells, and evaluation of their activation status have become the significant factors in the distinction of AR and NAR subtypes, including NAR with eosinophilia syndrome (NARES). Not only does this multiparametric approach improve diagnostic accuracy, but it also assists in the creation of an individualized treatment approach reflecting differences between inflammatory profiles, representing a substantial advancement in precision diagnostic approaches compared with conventional cytological assessment.

#### 4.1.2 Local specific IgE and rapid detection of inflammatory mediators

Local sIgE in nasal secretions or lavage fluid is a distinctive marker of nasal allergic inflammation without systemic atopy and has made LAR an important diagnostic consideration. In contrast to classical AR, the systemic sIgE production is observed in the nasal mucosa, whereas the systemic sIgE production is positive in LAR patients. Local sIgE can be detected following NPT or natural exposure to allergen and this has certain diagnostic implications to LAR. Nasal sIgE against HDM allergens, such as Dermatophagoides pteronyssinus and Dermatophagoides farinae, has also been shown to be extremely elevated in LAR patients compared with non-allergic controls, and related to the severity of symptoms and nasal provocation reaction (71). Nasal sIgE is also found in LAR, which supports the localized IgE production concept, which is also supported by the presence of IgE+ plasmablasts and IgE sequential class switch recombination in the nasal mucosa (72). This local IgE response reflects the same underlying type 2 inflammatory mechanisms described above, but occurring predominantly at the mucosal level rather than systemically.

Besides sIgE, the real-time analysis of the inflammatory mediators in nasal secretions enables dynamic information on the Th2-type activity in the nasal mucosa. Cytokines (IL-4, IL-5 and IL-13) and mediators (tryptase, leukotrienes and eosinophil cationic protein) can be determined in nasal lavage fluids and secretions and this is an indication of the local inflammatory state (73). Not only do these mediators confirm active allergic inflammation, but also clinical manifestations and mucosal changes are linked to them. To illustrate this, IL-5 is associated with the recruitment and activation of the eosinophil, IL-4 and IL-13 with IgE switching of classes and hypersecretion. These mediators are dynamic and can be measured and thus serve as a disease activity and therapeutic response biomarker.

Technological advances have facilitated the development of rapid and bedside diagnostic devices to detect local sIgE and inflammatory mediators. The quantification of nasal sIgE and cytokines in point-of-care can be done by using lateral flow immunochromatography and microfluidic chip-based tests as an alternative to the traditional laboratory-based methods, which are non-invasive, fast and dependable (43). These instruments are intended to address the limitations of nasal allergen provocation tests, which, regardless of being considered as the gold standard in the diagnosis of LAR, are time-consuming and require the skills of experts. This rapid assay type should be brought into clinical practice to enhance the precision of phenotyping rhinitis and allow more specific management to be created, including AIT to deal with local allergic reactions.

The concept of local IgE production remains debatable, though. The nasal sIgE in the NAR patients or healthy controls has not been consistently demonstrated in some studies, challenging the prevalence of LAR reported in certain populations (74). However, the diagnostic performance of nasal sIgE remains inconsistent across studies, with reported sensitivity and specificity varying widely depending on allergen type, assay methodology, and timing of sample collection. This variability limits its reliability as a standalone diagnostic tool. Moreover, low-level nasal IgE has been detected in some non-allergic individuals, raising concerns regarding false-positive results and potential overestimation of LAR prevalence (73). Therefore, nasal sIgE should be interpreted cautiously and preferably in combination with NAPT and clinical assessment. Therefore, nasal sIgE testing with nasal provocation testing (NPT) and identification of inflammatory mediators are a comprehensive approach to the adequate diagnosis of LAR.

**Figure 2:**
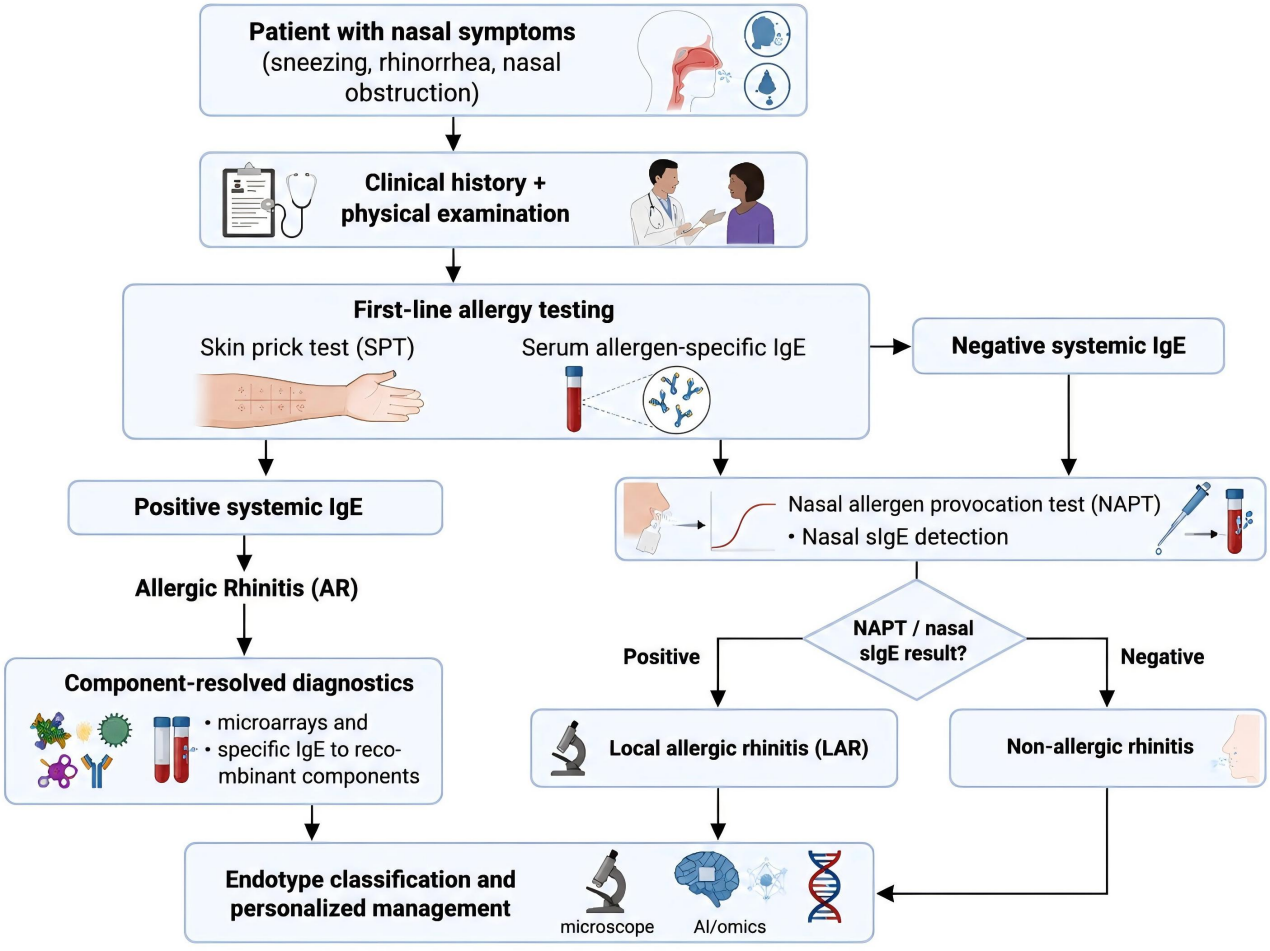
Integrated diagnostic workflow for allergic rhinitis, local allergic rhinitis, and non-allergic rhinitis.initial evaluation includes clinical history, physical examination, and first-line allergy testing (SPT or sIgE). Patients with persistent symptoms despite negative systemic sensitization may undergo nasal allergen provocation testing and local nasal IgE assessment to identify LAR. Advanced molecular and inflammatory profiling may further support personalized diagnosis and management.

In conclusion, the local sIgE and inflammatory mediators of nasal secretion are a significant step towards the diagnosis of LAR. It provides additional insight into localized inflammatory responses that involves Th2 in the background of LAR, which is not comparable to NAR or systemic AR. “The development of rapid point-of-care assays may facilitate earlier and more accessible diagnosis” thereby enabling more targeted treatment strategies based on local immune activity. An integrated diagnostic workflow incorporating both systemic and local immune assessments is illustrated in Figure 2.

### 4.2 Nasal provocation test

#### 4.2.1 Precision in allergen-specific nasal provocation testing

NPT is widely regarded as a reference standard in the diagnosis of AR, particularly when the clinical history and *in vitro* testing, such as SPT or serum-specific IgE (sIgE) testing, are inconclusive or discordant. As discussed in Section 2, conventional tests such as SPT and sIgE may not fully reflect clinically relevant sensitization, which underscores the complementary role of NPT. This is particularly relevant when testing new or less commonly encountered allergens or in suspected LAR, a form of allergy where the patient presents with an allergic reaction in the nose and not other areas of the body. Recent years have seen substantial improvements in the accuracy of NPT with methodological advances that have circumvented some of the previous limitations that were related to the technique in the form of allergen dosing, endpoint selection and objective measurement of nasal responses.

Traditionally, NPT has relied heavily on subjective symptom scoring, which, despite its clinical informativeness, has introduced variability and potential bias. Recent efforts have focused on the standardization of doses of allergens, objective endpoints, including nasal airflow measurements (e.g., peak nasal inspiratory flow, PNIF), acoustic rhinometry, nasal nitric oxide (nNO) levels, and nasal sound. These objective parameters provide quantifiable and reproducible data which supplements subjective symptom scores, and thereby increases diagnostic accuracy and reliability. An example is metered allergen application devices, which can deliver the allergen consistently to reduce the inter-test variability and enhance comparability between studies and clinical settings (42).

Its diagnostic and mechanistic utility has also been expanded by the continuous monitoring of inflammatory mediators that are released during NPT. Pre- and post-allergen challenge of local nasal sIgE and cytokines and other inflammatory markers can be used to measure dynamic evaluation of mucosal immune activation. This approach has been vital in the characterization of LAR whereby systemic markers are usually normal, but local IgE synthesis and mediator discharge is suggestive of allergen-specific nasal inflammation (43, 71). Hence, NPT is now not only a symptom-provocation test, but also a useful approach for investigating early- and late-phase allergic responses.

Available evidence suggests that NPT plays an important role in the differentiation between AR and NAR and LAR and in polysensitized individuals, who may have multiple allergens involved. The combination of sIgE to major allergenic molecules with NPT outcomes is more effective in predicting clinically relevant sensitizations in the selection of AIT in seasonal AR in pediatric patients (75). Similarly, the clinical significance of NPT among the sensitized to HDM patients is evidenced by borderline sIgE levels or wheal sizes of SPT, which may assist in identifying appropriate candidates for AIT (76).

NPT is also relevant in occupational AR whereby it is extremely important to diagnose the perpetrating allergens such as storage mites or bovine dander. The extracts of allergy tests and self-prepared extracts have been tailored to increase sensitivity and specificity in diagnostic testing with NPT is often used as a confirmatory tool for assessing clinical relevance (77, 78). In addition, NPT has been applied to determine the treatment effect including sublingual immunotherapy (SLIT) by objectively measuring changes in nasal reactivity and symptom scores over time as an objective endpoint in clinical trials and treatment monitoring (79).

Despite its advantages, NPT should be applied carefully with appropriate patient selection and standardized procedures to minimize the risks and ensure that it can be reproduced. Such warnings as serious asthma or anatomical defect of the nose should be considered. An ensemble learning model in combination with AI has been mentioned to assist in the interpretation of more complex clinical and laboratory data which could be utilized to optimize the utilization of NPT and reduce unnecessary tests (80).

#### 4.2.2 Non-specific nasal provocation tests and assessment of nasal hyperresponsiveness

Non-specific hyperreactivity of the nasal mucosa is a feature of AR, and is evaluated by the non-specific NPTs using histamine or acetylcholine as provoking agents. Unlike the allergen-specific NPTs, which detect the allergen-specific responses, non-specific NPTs expose the nasal mucosa to pharmacologic agents that trigger nasal symptoms through direct stimulation of the sensory nerves or vascular structures. It is one way of obtaining an objective measure of NHR. Histamine is a well-characterized mediator, which is discharged in allergic inflammation and leads to nasal congestion, sneezing, and rhinorrhea via the H1 receptors. Acetylcholine is a parasympathetic neurotransmitter which leads to glandular secretion and vasodilation via muscarinic receptors. Intranasal delivery can replicate the nasal effects of these agents and induce reproducible nasal symptoms and measurable nasal airflow resistance changes that may be used to measure mucosal hyperreactivity.

The clinical utility of non-specific NPTs lies in their ability to objectively assess NHR, which has been linked to severity of disease and symptom burden in AR patients. Mucosal inflammation and neural sensitization manifested by high levels of mucosal responsiveness to histamine or acetylcholine that can be sustained even in the absence of allergens. This hyperreactive state that is linked with symptom exacerbation due to non-allergic triggers, such as irritants, changes in temperature, or infections. Clinicians will be able to classify patients according to the intensity of their nasal hyperreactivity by determining the dose/concentration of histamine or acetylcholine required to produce nasal symptoms or airflow blockage. The stratification can be used to forecast the disease course, personalize pharmacologic therapy, and evaluate the treatment outcomes.

Non-specific NPTs are also helpful in differentiation of the subtypes of rhinitis. For example, the nasal responsiveness of AR patients is generally higher in comparison to healthy individuals or with patients of NAR. However, there are also other forms of NAR subtypes such as NAR with the eosinophilia syndrome (NARES) which may also be more nasal hyperreactive. This points to the importance of non-specific NPT result interpretation alongside the clinical history and other diagnostic instruments. Non-specific NPTs are used to complement allergen-specific testing by providing objective evidence of NHR measurement to provide information on the functional status of the nasal mucosa in addition to allergen sensitization.

Non-specific NPTs have been very important in the research settings in explaining the pathophysiological mechanisms of NHR. It has been found that repeated exposure to allergens and chronic inflammation pre-disposes mucosal sensitivity to non-specific stimuli by increasing the number of sensory nerve receptors and the release of neuropeptides. These neurogenic inflammation maintains the hyperreactive phenotype, which contributes to persistent symptoms. In addition, non-specific NPTs have been employed to determine the efficacy of such therapeutic interventions as antihistamines, intranasal corticosteroids, and allergen immunotherapy. Non-specific NPTs are used as objective outcome measures as clinical improvement was linked to a reduction in nasal responsiveness following therapy.

Although NPTs would be beneficial, non-specific NPTs would require standardized protocols to be reproducible and safe. There should be clear choice of provoking agent, concentration increments, mode of administration and positive response criteria. The one most commonly used is histamine since it has been well-characterized and is available, although acetylcholine and other agents, such as methacholine have been used. Objective tests (nasal airway resistance (NAR) by rhinomanometry or acoustic rhinometry) are used along with subjective symptom scoring to measure symptom responses. Contraindications such as severe asthma or cardiovascular disease should be included in the risk reduction.

In conclusion, non-specific NPTs containing histamine or acetylcholine is an effective and objective way of assessing nasal mucosal hyperresponsiveness, which is a major feature of AR. The tests are applicable in establishing the severity of the disease, in the decision to be made in treatment and in differentiating the subtypes of rhinitis by measuring the degree of hyperreactivity. They assist in enhancing the precision of diagnosis and treatment of rhinitis as they will be incorporated in clinical practice and research, which will assist in the transformation of the traditional method of diagnosis to a more advanced, individualized diagnosis method. The role of non-specific NPTs in the overall assessment of allergic and non-allergic nasal illnesses will be additional developed with the testing guidelines and additional clinical implementation (81, 82).

### 4.3 Other inflammatory and functional markers

#### 4.3.1 Nasal airflow function and aerodynamic assessment

##### 4.3.1.1 Objective measurement of nasal airflow

As part of objective measurement of nasal obstruction, which is a major symptom of AR and other nasal pathologies, nasal airflow and aerodynamic assessment is necessary. It has been demonstrated that nasal cavity volume, minimum cross-sectional area and total nasal resistance can be reliably measured with active anterior rhinomanometry (AAR) and acoustic rhinometry in addition to subjective ratings of nasal congestion. AAR is a direct measure of NAR that measures the pressure and airflow during the process of breathing, which is an objective correlate of the clinical severity of nasal obstruction. It has been demonstrated that both moderate and severe AR patients demonstrate significantly high NAR values in comparison with patients with mild disease which are in accordance with the AR and its Impact on Asthma (ARIA) system of severity grading (83). This objective measure is not only a measure of nasal obstruction but also an indicator of at-risk patients who might have latent lower airway involvement in that high nasal resistance was associated with bronchial hyperreactivity even in the absence of overt asthma symptoms. It is based on this that AAR can be utilized as a diagnostic and risk stratification helpful tool in AR.

##### 4.3.1.2 Acoustic rhinometry and functional assessment

An addition to the rhinomanometry, acoustic rhinometry measurements has additional functional information about vibratory properties of the nasal airflow which can be utilized to find out whether there are mucosal congestion and structural abnormalities. The methods used to measure nasal patency can accurately determine the condition compared to symptom scores to assist clinicians modify therapeutic interventions.

##### 4.3.1.3 Computational fluid dynamics and advanced imaging

In addition to these routine functional measurements, the interaction of computational fluid dynamics (CFD) with high-resolution imaging, e.g., computed tomography (CT) or magnetic resonance imaging (MRI) has substantially improved the knowledge of airflow patterns in the nose, the anatomic and physiologic determinants of the patterns. The CFD can be used to reconstruct and model the nasal airflow, pressure distribution, and wall shear stress at different breathing conditions in three dimensions. The mechanism explains the complicated interaction between nose anatomy, including septal deviations, turbinate hypertrophy and nasal valve collapse and the airflow dynamics, which is the reason why people experience nasal blockage (84). Using the example of CFD analyses, patients with septal deviation and AR have been displayed to be experiencing a dissimilar velocity contour and an augmentation in airflow resistance in contrast with standardized nasal models which depict the functional effects of anatomical deviations.

Additionally, the CFD has also been utilized when assessing the aerodynamic implications of surgical procedures that can be utilized in the treatment of nasal blockage. Relative evaluation of poorer methods of inferior turbinate resection like total inferior turbinectomy and turbinoplasty have portrayed slight variations in the post-surgery NAR and airflow pattern. Turbinoplasty can also maintain a mucosal surface and the airflow profiles are more likely to resemble normal controls and total turbinectomy can result in high-energy jet-like airflow inferiority, which can disrupt nasal air conditioning and mucosal wellbeing (85). The insights are important to the surgical planning and allow individualized planning with the highest number of positive effects on airflow improvement and maintenance of nasal physiology.

##### 4.3.1.4 Clinical applications and personalized management

These sophisticated tests play a clinical role in managing nasal obstruction syndrome (NOS) which is a multifactorial disease made up of anatomical, inflammatory as well as functional factors. The objective assessment of the airflow resistance of the nose and aerodynamics patterns can help to determine which of the structural abnormalities, mucosal edema, and neurosensory dysfunction play its roles in nasal obstruction. The general knowledge can guide multidisciplinary treatment, e.g., pharmacologic (e.g., corticosteroids, antihistamines), immunotherapy, and surgery according to anatomical features and pathophysiology of the disease (86).

Moreover, the nasal airflow testing is not limited to nasal symptoms, and its obstruction can negatively influence the olfactory, sleep quality, and general respiratory health. For example, when the mucosal origin swellings and congestion become indicative of pregnancy rhinitis, the nasal airflow and olfactory sensitivity might be lost in the patients who are not conscious of the losses subjectively, which can be objectively measured by the rhinomanometry and olfactory tests (87). These findings support the incorporation of objective nasal airflow assessment in a broader range of clinical settings (88).

Overall, AAR and acoustic rhinometry are a solid and objective NAR and patency measure that supplements the subjective symptom measure in AR and other related diseases. The integration of CFD and high-resolution imaging enables three-dimensional visualization and simulation of nasal airflow (83), and that explains the anatomic and functional basis of nasal obstruction. This form of high-tech diagnostic modalities contributes to a more comprehensive understanding of nasal physiology and pathophysiology, individual surgical planning, and accurate measurement of the therapeutic outcome but also represents a substantial advancement over conventional diagnostic approaches for nasal airway disorders.

#### 4.3.2 Refined assessment by nasal endoscopy and imaging

The introduction of high-definition electronic nasal endoscopy, the clinical examination of AR has improved clinical assessment of AR by enabling a close-up view of the nasal mucosa, turbinates, and secretions. The technology allows clinicians to detect subtle changes in mucosal color, edema extent, and the character of nasal secretions that are significant indicators of inflammatory diseases. An example of this is the inferior turbinates in AR patients, which normally have typical color changes, i.e., pale and edematous color changes that transform into erythematous and inflamed color changes, which are the signs of underlying allergic inflammation and vascular congestion. Nasal endoscopy dynamic assessment is also an efficient method of monitoring the nasal cycle and responsiveness to pharmacologic agents, and is a method of giving real-time feedback on the effectiveness of the therapy. The results of recent studies based on deep learning algorithms to classify nasal endoscopy images have been shown to be good diagnostic, with models achieving up to 90.8% accuracy in detecting AR-related mucosal changes by counting the color distribution of the inferior turbinate region in the CIE-Lab color space. The specified quantitative technique is an objective, non-invasive addition to the conventional clinical check-up that can potentially reduce the application of invasive SPT and enhance the precision of diagnosis (88). Nasal endoscopy should also nasal endoscopy should be applied to help differentiate between AR and other diseases with similar symptoms like nasal myiasis or chronic rhinosinusitis, by observing the pathological alterations first hand, i.e., larvae or polyps, and, consequently, the appropriate treatment (89). Together with the scores of clinical symptoms and allergen testing, endoscopic results are closely related to the severity of the disease and response to therapy, and this fact contributes to their significance in the general assessment of patients (80). In addition to this, anatomical differences and mucosal changes that may predispose the patients to complications, such as sinu barotrauma, can be detected using nasal endoscopy, and a comprehensive endoscopic study should be conducted in at-risk groups (90). Nasal endoscopy may be used in the diagnosis as well as in the therapeutic monitoring. Studies have found out that significant changes in the mucosal appearance and the decrease in edema following SLIT with modified Lund-Kennedy scores are good objective measures of the changes that are associated with symptom relief (91, 92). Furthermore, portable and affordable endoscopic devices have been developed, which has enhanced the access of nasal endoscopy in primary care and resource-limited settings, enabling nasal pathologies to be identified and treated at an early phase (93). Combined with emerging AI technologies, high-definition nasal endoscopy represents a promising advancement in the correct and dynamic assessment of the nasal mucosa condition in AR to take timely and personalized measures.

New imaging technologies, such as optical coherence tomography (OCT) and advanced CT, have emerged as valuable adjunctive tools for non-invasive, high-resolution evaluation of nasal mucosal microstructure to complement endoscopic evaluation. With OCT, in particular, cross-sectional imaging is provided, which can outline the epithelial layer and superficial lamina propria, thus making it possible to measure the mucosal thickness, edema, and tissue remodeling without a biopsy. This aspect is particularly important in AR, in which structural changes and mucosal edema contribute to the severity of symptoms and disease progression. Although OCT is still a young technology in its clinical application, it has proven capable of detecting small mucosal alterations that are linked to inflammatory events, as a disease severity biomarker and a predictor of therapeutic response. The reference standard for evaluating sinonasal anatomy and pathology is high-resolution CT imaging that gives a close-up view of the bony structures, sinus opacification, and polypoid lesions. Mucosal thickening of the inferior and middle turbinates and medial ethmoid area with little extension to the ethmoid area is common in CT of AR patients and is a localized pattern of allergic inflammation (94). The identification of anatomical variants such as concha bullosa, infraorbital ethmoid cells, and variations in the sphenopalatine foramen can also be determined with the assistance of CT imaging and have a great impact on the surgical planning and risk stratification (95). Imaging findings, together with endoscopic findings, may enhance the accuracy of the diagnosis, surgery, and recurrence of the illness, particularly CRS with nasal polyps, where the imaging scores, such as Lund-Mackay, are linked to clinical outcome (96, 97). Surgical navigation is also improved by the use of new techniques of three-dimensional reconstruction and intraoperative imaging in real-time, which improves the accuracy and safety of endoscopic sinus surgery (ESS) (98). These imaging modalities facilitate a transition toward more quantitative and objective diagnostic assessment of the quantitative, objective diagnostics and the obsolete qualitative evaluation techniques, enabling the application of precision medicine and informing the approach to individual treatment.

### 4.4 Exhaled and nasal nitric oxide detection

#### 4.4.1 FeNO

##### 4.4.1.1 FeNO and united airway disease

Fractional exhaled nitric oxide (FeNO) is a widely recognized biomarker reflecting type 2 airway inflammation, as outlined in the general immunological framework above, and provides a non-invasive measure of ongoing allergic inflammatory activity. However, elevated FeNO levels are also typical of AR patients, highlighting its usefulness as a convenient biomarker for measuring systemic allergic inflammation and the existence of upper and lower airway diseases. United airway disease as a concept is based on the fact that the upper and lower respiratory tract are pathophysiologically and clinically linked such that inflammation in either of the compartments will be likely to be reflected or influenced by the other. Thus, the FeNO is a non-invasive, real-time measure of airway inflammation, which is anatomically across anatomical compartments and reflects the aggregate of the inflammatory state of the respiratory system. When interpreted alongside local biomarkers described in Section 4, FeNO contributes to a more integrated assessment of airway inflammation.

Clinical studies have shown that even without the manifestation of lower airway symptoms, the level of FeNO of AR patients is significantly elevated, which is evidence of subclinical lower airway inflammation or systemic Th2 inflammation. As an example, AR patients with comorbid asthma have higher levels of FeNO compared to healthy controls and these levels are linked to disease control and eosinophilic inflammatory markers such as blood eosinophil counts (99). Serum periostin, which is another Th2 biomarker, is also positively correlated with FeNO, which justifies its presence in the representation of systemic allergic inflammation (100). These findings support the use of FeNO as a surrogate endpoint to ascertain the overall airway inflammatory load and patients who are likely to have lower airway involvement and guide the overall management strategy (99).

##### 4.4.1.2 Clinical utility of FeNO

To avoid contamination and ensure the accuracy and clinical usefulness of FeNO measurements in AR and asthma, standardized sampling protocols are necessary since nasal NO is generated in large concentrations in the upper airways and may artificially elevate the FeNO measurements. FeNO is a biomarker and requires standardized tests like control of the exhalation flow rate and the means of eliminating nasal NO contamination to ensure that FeNO is reliable in the management of AR (101). Such standardization allows clinicians to distinguish between upper and lower airway sources of NO, which increases the accuracy of the diagnosis and monitoring of airway inflammation.

Additional potential is evident by the use of FeNO in comorbidities like obstructive sleep apnea (OSA) because high FeNO levels are correlated to the severity of the disease, which shows that there is a similarity in the inflammatory mechanisms of upper and lower airway diseases (102). These environmental factors can include the exposure to particulate matter and microbial elements in indoor and outdoor settings and it is important to note the environmental modifiers when analyzing the results in AR patients (103, 104). These findings demonstrate the multifaceted nature of airway inflammation and the need to integrate the measurements of FeNO with the clinical and environmental assessment.

##### 4.4.1.3 FeNO in pediatric and comorbid conditions

FeNO is established as a useful biomarker of allergic airway inflammation in the pediatric population, and the higher the level of FeNO, the greater the allergic sensitization, symptoms severity, and response to treatment in children with AR and asthma (105). The FeNO, when combined with nasal NO (FnNO), enhances the examination of airway inflammation and supplements the data on the upper and lower airway involvement (106, 107). The integrated approach contributes to the acceptance of the concept of united airway disease and allows individual therapeutic responses.

#### 4.4.2 nNO

##### 4.4.2.1 Physiology and measurement of nNO

The principal producers of nNO are epithelial cells covering the nasal mucosa and paranasal sinuses, namely, ethmoid and maxillary sinuses. The production and release of nNO by the nasal airway has been well studied and parameters, especially by the use of nasal sampling, have been standardized to ensure reliability and clinical utility. Physiologically, nNO assists in the control of local vascular tone, mucociliary clearance, and antimicrobial defense which are all critical to the health of the nasal mucosa. When the patient is put through breath-holding or controlled breathing maneuvers, the chemiluminescence or electrochemical analyzers tend to standardize the measurement of nNO, which enables non-invasive examination of nasal airway inflammation and functioning (108, 109).

##### 4.4.2.2 nNO in allergic rhinitis

The level of nNO is different in AR particularly during acute exacerbation. Studies have reported elevated levels of nNO in AR patients compared to healthy controls, which is symptomatic of eosinophilic inflammation and Th2 cytokine environment that is characteristic of allergic airway disease. To illustrate, children with moderate-to-severe persistent AR have significantly greater amounts of nNO, which are positively correlated with peripheral eosinophil counts and total serum IgE, suggesting that nNO can be employed as a surrogate endpoint of upper airway eosinophilic inflammation (110). Such interventions as intranasal corticosteroids and antihistamines reduce nNO levels, as do clinical improvement and the reduction of inflammatory outcomes, but nNO is not necessarily a reliable indicator of treatment response in the absence of other biomarkers such as FeNO or symptom scores (111, 112).

##### 4.4.2.3 nNO in chronic sinonasal diseases

Along with AR, nNO measures are also very important in the diagnosis of chronic nasal and sinus inflammatory diseases. CRS and chronic rhinosinusitis without nasal polyps (CRSsNP) in particular is often associated with a reduced level of nNO due to obstruction or inflammation of the sinu ostia, and therefore preventing the diffusion of NO to the nasal cavity. Nevertheless, on the contrary, primary ciliary dyskinesia (PCD) is a genetic disorder linked to defective ciliary motility, which has a strong low nNO levels, which is typically considered as a screening and diagnostic instrument. The nNO of PCD is extremely low, a quality that suggests the disturbed release or production of NO due to the dysfunction of the cilium and sinus hypoventilation (109). Thus, nNO is a good non-invasive technique of distinguishing between the subtypes of CRS and the diagnosis of PCD, which can be used to define the further diagnostic work and treatment.CRSwNPs have a tendency to have lower levels of nNO than healthy individuals, which is a sign of sinus ostial obstruction and mucosal inflammation. CRSwNP patients who are eosinophilic respond well to nasal corticosteroid treatment and exhibit elevated levels of nNO following ESS and the levels go back to normal with inflammation remission. Non-eosinophilic CRSwNP patients, conversely, tend to possess a continuously low nNO and reduced steroid responsiveness, proving the potential of nNO in endotyping CRS and predicting its treatment (113). In addition, asthma patients who are comorbid with nasal polyposis are more likely to have a higher nNO level compared to patients not having polyps, which is an indication of systemic type 2 inflammation both in the upper and lower airways (114).The distribution of nNO and its dynamics in the nasal cavity is influenced by anatomical and physiological factors. CFD has explored the nNO concentrations and they have been found to be affected by the velocity of the airflow, the sinuosity of the ostium, and diffusion. The release of NO is mainly by the paranasal sinuses, more so by the ethmoid sinuses, into the nasal cavities and secondary is by the convection. The alterations in the ostiomeatal complex could lead to a significant alteration in the levels of nNO, which is significant in the surgical reconstruction to regain the normal sinus ventilation and distribution of NO (109, 115). These biomechanical results suggest the importance of the nasal anatomy factor when explaining nNO measurements in clinical practice.

##### 4.4.2.4 Limitations of nNO as a biomarker

Despite the potential of nNO use as a biomarker in AR, the role of nNO as an independent biomarker in AR is controversial. Other researchers have not demonstrated any significant differences in the degree of nNO between allergic and NAR patients and this implies that nNO should be applied alongside other clinical and laboratory tests to draw the right diagnosis (116). In addition, the nNO levels may not be directly connected to the pulmonary functioning or the management of asthma, and thus they should be assessed alongside the FeNO and other inflammatory indicators to give a complete airway assessment (101).

## 5 Emerging and integrative technologies

Recent advances have introduced novel technologies that integrate clinical, imaging, and molecular data for more comprehensive analysis.

### 5.1 Omics technologies and systems biology in diagnosis

#### 5.1.1 Transcriptomics and epigenetics in identifying diagnostic biomarkers

The integration of both transcriptomic and epigenetic research has also made a significant contribution to the research of the pathogenesis of AR and has helped to determine novel diagnostic biomarkers. AR-specific gene expression patterns are fully defined by transcriptomic profiling, particularly by RNA sequencing of nasal mucosa brushings or biopsy samples. This has always been observed in the upregulation of the genes of Th2 pathways, including IL5RA, IL1RL1 and CST1, which are the major mediators of type 2 inflammation that is characteristic of AR (117, 118). It is also worth mentioning that CST1, a gene that codes cystatin SN, is now a potent biomarker, as its expression is higher in upper and lower airways of AR patients and it also correlates with Th2-high asthma phenotypes, which explains the concept of united airway disease (117, 119). Besides the discovery of Th2-driven signatures, transcriptomic analyses have demonstrated patterns of gene expression that might be utilized to determine AR compared to other allergic diseases and reveal multimorbidity signatures in the form of eosinophil-associated immune responses and IL5/JAK/STAT signaling pathways (118, 120). In addition, single-cell RNA sequencing has refined this concept and has defined immune cell subsets and their transcriptional dynamics in AR, including the state of B cell differentiation and the role of integrin beta-1 (ITGB1) in the activity of memory B cells. These findings can not only enhance mechanistic knowledge of the AR pathogenesis, but also indicate the potential biomarkers and treatment targets of precision medicine strategies (121).

Another regulatory level that influences gene expression in AR is epigenetic modifications including DNA methylation and histone modifications and may provide a promising approach of early risk identification and correct diagnosis. DNA methylation profiling of nasal epithelial cells and peripheral blood mononuclear cells has revealed the differentially methylated regions (DMRs) of AR susceptibility and disease severity. The gene-environment interactions that regulate immune responses are also reflected in the methylation patterns of the genes such as IL5RA, SIGLEC8 and FOXP1 that differ between allergic phenotypes (122, 123). It has also been reported that long non-coding RNAs (lncRNAs) play a role in the epigenetic control of immune homeostasis. The expression of certain lncRNAs is related to the microbiota composition and the risk of allergies, which means that they can be used as biomarkers that can mediate environmental exposures and genetic predisposition (124). Associated genes, such as SIN3A, have been proposed to have a central role in immune cell differentiation, and specifically in Th17 to Tregs balance, which contributes significantly to the pathogenesis of AR (125). In addition, m6A RNA methylation is a novel epigenetic pathway, which controls such inflammatory pathways as MAPK in the nasal mucosa, and enzymes such as ALKBH5 are possible biomarkers and therapeutic targets (126). Combined, these epigenetic results are expanding the definition of AR pathophysiology, new molecular pathways defining disease susceptibility, severity, and sensitivity to treatment, and offering future opportunities of molecularly guided diagnostics and customized treatment.

The combination of transcriptomic and epigenetic alterations indicates the complexity of AR and the necessity of multi-omics methods in the context of finding biomarkers precisely. Integrated analyses of gene expression, DNA methylation, and non-coding RNA have shown molecular pathways that mediate AR including Th2-biased immune responses, epithelial barrier dysfunction, and neuroimmune interactions (123, 127). These molecular signatures not only help to diagnose the patient in the early stages, but also enable the patient to be stratified to receive customized treatment, including allergen immunotherapy, which has been shown to modulate transcriptomic profiles to an immune tolerance state (128, 129). In addition, accelerated epigenetic age has been identified to be associated with allergic diseases, including AR, and there is potential of using epigenetic biomarkers as a disease load and disease progression marker (130, 131).

Overall, the transcriptomic analysis of nasal mucosa samples has shown AR-specific gene expression, particularly Th2-related genes, that are diagnostic and phenotypic biomarkers. Simultaneously, cellular epigenetics suggests DNA methylation and histone modification patterns, which mediate gene-environment interactions and immune regulation in AR. Integrating these layers of omics has the potential to develop precise non-invasive diagnostic techniques and guide therapeutic treatments, which may contribute to the development of precision medicine approaches in AR.

#### 5.1.2 Proteomics and metabolomics analysis of body fluid samples

The proteomics of body fluids and metabolomics of serum and nasal secretions have proven useful in elucidating the pathophysiology of AR and determining unique molecular fingerprints that would be utilized to diagnose and treat the disease in a more precise and personalized manner. Using more advanced mass spectrometry (MS)-based technology, including liquid chromatography-tandem MS (LC-MS/MS) and nuclear magnetic resonance (NMR) spectroscopy, researchers have completely profiled the proteome and metabolome of numerous biofluids and have found certain patterns associated with AR pathophysiology. An example is multi-biofluid metabolomics of children with respiratory diseases caused by allergies showed that metabolites in blood, stool, and urine samples changed significantly and the metabolic profile in blood samples could diagnose AR with the highest diagnostic accuracy (area under the curve [AUC] = 0.732) compared to other biofluids (132). These findings highlight the systemic metabolic disturbances that go hand in hand with AR and lead to the potential of blood-based metabolite panels as non-invasive biomarkers.

Proteomic analyses are employed to complement metabolomics in the discovery of differentially expressed proteins that play a role in immune and inflammatory pathways that form the central focus in AR. The analysis of the traditional Chinese medicine formulations to treat AR by integrated proteomics and network pharmacology showed that the proteins (MAPK1, SYK, and Rac1) are implicated in the signaling cascades, PI3K-Akt and FcereRI, which mediate allergic inflammation (133). They are used as therapeutic targets and disease activity and treatment response candidate biomarkers. Besides, proteomic studies of AR models of nasal mucosal tissues showed that cell adhesion molecules including E-selectin, VCAM-1 and ICAM-1, which are important in the recruitment of leukocytes and inflammation, were altered, which directly correlates with proteomic changes and the pathogenesis of AR (134).

Proteomics and metabolomics data have been combined, and now it is easier to identify biomarker combinations that can differentiate between AR sub types, predict the success of immunotherapy, and monitor the course of the disease. For example, metabolomic studies have also determined the significance of short-chain fatty acids (SCFA) such as butyric acid and acetic acid that are microbial-produced metabolites negatively correlated with serum IgE levels and allergen sensitization in AR and asthma (132). These metabolites reflect gut microbiota-host interactions that regulate systemic immune responses and that metabolite signatures can be used as disease endotype predictors and therapeutic targets. Proteomic analyses have also indicated that the regulation of endoplasmic reticulum stress-related proteins and glycolysis pathways are linked to inflammatory conditions and symptom severity in AR and provide mechanistic data and possible biomarkers to evaluate in the clinical practice (135).

The recent advances in high-throughput MS techniques have increased low abundance protein and metabolite detection in complex biofluids, enhancing sensitivity and specificity of AR biomarker discovery. For example, UHPLC-HRMS has enabled the identification of dozens of bioactive compounds and their protein targets in herbal medicine studies to elucidate multi-target effects and give new molecular signatures of therapeutic responses (134). Molecular docking and validation assays are another proof that these targets are useful biomarkers in the diagnosis and treatment monitoring of AR.

Concisely, proteomics and metabolomics of serum and nasal secretion provide the complete picture of the molecular environment of AR and show characteristic protein and metabolite fingerprints, which suggests heterogeneity of the disease and immune dysregulation. To prove these biomarkers and transform them into clinical routine to improve AR care, the future research that integrates multi-omics data with clinical phenotyping and longitudinal research will be necessary.

### 5.2 Integration and application of AI and digital health technologies

#### 5.2.1 Machine learning diagnostic models based on medical imaging and clinical data

The integration of ML algorithms and medical imaging and clinical data has introduced a new era of diagnosis of AR with more precise, objective, and consistent outcomes. One of the most promising directions is the analysis of nasal endoscopy images using deep learning algorithms. Nasal endoscopy can be used to directly visualize the nasal mucosa, and typical AR inflammatory features such as mucosal pallor and edema can be seen. The traditional diagnosis is highly reliant on the subjective perception of the clinicians and as such, it is prone to inter-observer error as well as influenced by the experience of the clinician. Algorithms of deep learning particularly convolutional neural networks (CNNs) have demonstrated that they can detect subtle features in images that are associated with these mucosal alterations. For example, a study of the color distribution of the inferior turbinate area in nasal endoscopy images utilized the CIE-Lab color space as a measure of mucosal pallor and edema. The model demonstrated promising accuracy in the diagnosis of AR with a diagnostic accuracy of approximately 90.8 percent when extracting adaptive histogram features and a combination of CNN-based feature extraction and support vector machine (SVM) classifier (88). It is a non-invasive image-based quantitative technique that reduces the differences in diagnosis between physicians, and allows early screening and grading of the severity of the disease, which can be used to intervene early.

In addition to imaging, multidimensional clinical data, such as electronic health records (EHRs), allergen sensitization profiles, history of environmental exposure, and common laboratory tests in ML systems have also increased the accuracy of diagnosis and risk stratification. The ensemble learning models and these involve the combination of two or more classifiers to enhance the power and the quality have been effectively utilized to diagnose AR using the heterogeneous data sources. An adaptive random forest-out-of-bag ensemble (ARF-OOBEE) trained on clinical symptoms was more sensitive, specific and area under the receiver operating characteristic curve (AUC) than the traditional classifiers such as Naive Bayes, logistic regression and standard SVMs in a study (80). In the same manner, the parameters of routine blood tests (eosinophil counts, basophil levels, platelet indices, etc.) have been integrated into ML models, such as LightGBM and random forest, to build early screening tools of pediatric AR, and the AUC values are over 0.85 (136). These results indicate that the given laboratory data can play an important role in enhancing the diagnostic procedures without adding any extra load to the patients.

Integration of imaging and clinical data into ML systems supports of phenotyping and risk assessment on a case-by-case basis. They can be predicted by taking the data on environmental exposure, such as the pollen counts and air pollution index, and combining it with the clinical and immunological profile of a certain patient to rank them according to their risk of developing AR or getting an exacerbation. This interdisciplinary approach to the methodology adds to the individualized diagnosis prescription and management approach, and it is no longer the one size fits all paradigm. Besides, other respiratory diseases that share similar symptoms, such as diffuse panbronchiolitis, where AR was one of the most important diagnostic factors alongside radiological outcomes, have been examined with the assistance of ML models, which confirms that the technology can assist in reducing the range of diagnosis in complex clinical cases (137).

Despite these advances, there has been an issue with the application of the ML diagnostic models in the everyday clinical practice. The variation in image acquisition procedures, the variations in patient samples, and the necessity to work with big and well-labeled datasets restrict the extrapolability of existing models. Besides, explainability and interpretability are of paramount importance with respect to clinician acceptance and regulatory approval. Attempts to add explainable characteristics, e.g., the measurable values of mucosal color or anatomical measurements using segmentation, to the ML models have been promising to seal this divide (138). The ML tools can be supplemented by adding eHealth technologies, such as smartphone-based monitoring of the symptoms and digital patient-reported outcomes to deliver the real-time streams of data to monitor and streamline the model (139).

#### 5.2.2 Mhealth and wearable devices for symptom monitoring and trigger detection

The introduction of mHealth technologies and wearable devices into the process of AR management may be considered as one of the major steps towards monitoring of the symptoms and personal care. One of the most significant changes in the subject that allows patients to note the symptoms, medications, and exposure to allergens in an objective and continuous manner is the use of smartphone applications together with electronic diaries. An example is the symptom severity on visual analog scale (VAS) can be logged in real-time and this can offer a dynamic and detailed overview of disease variations that is superior to the conventional episodic clinical measures. The consistency of data collection improves the treatment plan compliance and enables health workers to apply interventions that would be specific to the needs of a particular patient. Control of AR and Asthma Test in Children (CARATKids) is such a validated scale, and it has been successfully translated into the mobile application form and proved to be comparable to the paper-based version in terms of reliability and internal consistency. The caregivers and children have demonstrated an ideal acceptance and preference to electronic administration which promotes remote monitoring and telehealth interventions in the pediatric population with AR and asthma (140).

The wearable devices are also used in real-time monitoring of allergen exposure in the environment as well as the symptom monitoring, which is required in dynamic risk assessment and personalized warning. The devices can be utilized in the determination of the pollen and other allergens concentration in the air and comparison of the environmental outcomes with the subjective nasal symptoms, including congestion, which can be evaluated by voice pattern alteration or nasal airflow blockage. The environmental detection and physiological observation can be interactively responded to and give a holistic picture of the precipitating factors and symptom exacerbations. A number of health applications of smartwatches and other wearable sensors have been explored, such as AR, to provide continuous health data and aid in self-management. Although the studies specifically on AR are yet to be conducted, the overall wearable health interventions suggest such benefits as improved symptom management, improved medication adherence, and reduced healthcare utilization (141).

AI-based digital health solutions can be added to the functionality of mHealth and wearable solutions to provide real-time and personalized recommendations based on symptoms and environmental exposures. Wearable sensors, electronic diaries, and patient-reported outcomes are very large datasets that can be analyzed using AI algorithms to forecast symptom flares and optimize treatment regimens. It has also been highlighted in systematic reviews that digital technologies, including mobile applications, telemedicine, and wearable devices, are very effective in symptom management and medication adherence of AR patients, and the symptom control improvements have been reported to be up to 82% and adherence rates up to 90%. These tools also ensure that the visits to the patient are less frequent and that the patient is more satisfied to have the opportunity to monitor the patient remotely and intervene in time (142).

Despite such promising advances, there remain concerns in the mass application of mHealth and wearable technologies to control AR. Such issues as device battery life, internet connection, compatibility of data across platforms and the pressure of having to manage many digital devices can affect the interaction of users and the quality of data. In addition, accuracy and clinical validation of environmental allergen sensors and symptom detection algorithms should be further researched to ensure that they are accurate and helpful. The second step of the evolution ought to be the integration of molecular biomarkers and digital technologies to make the diagnosis more precise and the treatment even more individualized. There is also the need to make sure that these technologies can be accessed and used by various categories of patients to maximize the benefits of these technologies in the daily clinical practice (141, 142). Current AI models are primarily trained on retrospective datasets. Validation is often limited to internal cross-validation or single-center cohorts, and external multi-center validation is sparse. Reported performance metrics such as accuracy, sensitivity, specificity, and AUC vary widely, emphasizing the need for standardized benchmarking (140). Many studies utilize relatively small datasets or incomplete clinical records, which may limit model generalizability. Biases in dataset composition (e.g., age distribution, geographic differences, or underrepresentation of rare phenotypes) can affect model predictions and clinical applicability (142). Translating AI models into routine practice requires addressing interpretability, integration with electronic health records, regulatory compliance, and clinician acceptance. Without clear workflow integration and prospective validation, the clinical impact of AI remains uncertain. In summary, while AI holds promise for automated pattern recognition, risk stratification, and predictive modeling in AR, these limitations must be carefully considered to avoid overestimating its current clinical utility.

In conclusion, the incorporation of the smartphone application, electronic diaries, wearable environmental sensors, and AI-driven analytics is transforming AR management. These technologies enable the continuous monitoring of symptoms and objective and dynamic evaluation of the risks, which give personalized care and improve patient outcomes. Research, clinical experimentation, and human-centered design will also have a major role in realization of the full potential of mHealth and wearable devices to improve the accuracy of diagnosis and treatment of AR.

**Figure 3:**
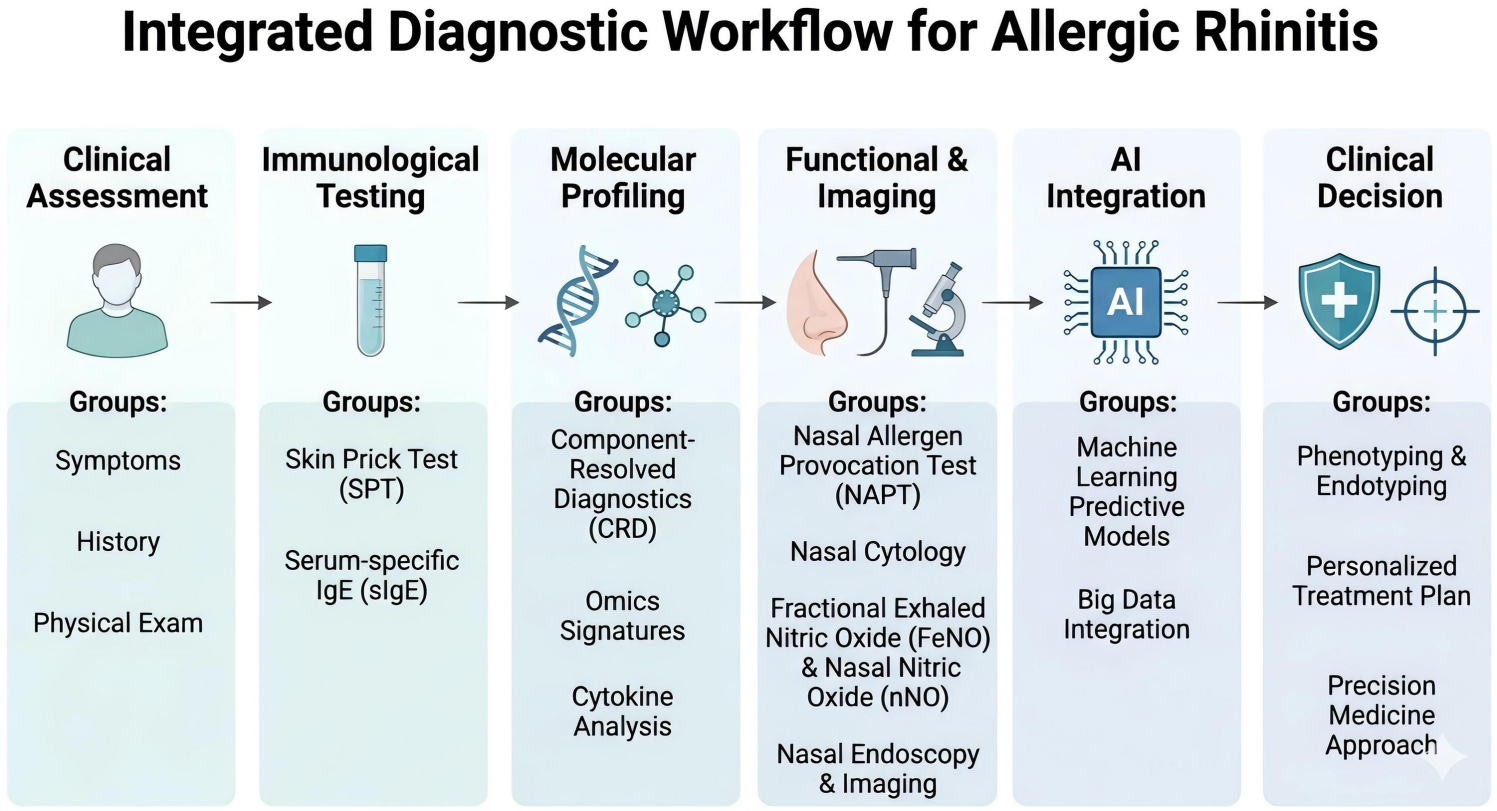
Workflow of integrated diagnostics for allergic rhinitis.

To illustrate how the various diagnostic modalities for AR can be applied in a coordinated manner, we propose an integrated diagnostic workflow (Figure 3). This stepwise framework incorporates traditional assessments, molecular evaluation, functional and biomarker-based testing, and emerging technologies, ultimately guiding clinical decision-making.

## 6 Advances in diagnostic strategies for children and special populations

### 6.1 Challenges and adapted techniques in diagnosing AR in children

The diagnosis of AR in children is associated with certain issues that demand a modification of the traditional ways of diagnosing the disease and the development of less invasive techniques. One of the greatest challenges is the low compliance of pediatric patients to conventional approaches, such as SPT and blood collection by means of venipuncture. These methods can be uncomfortable and painful with the result of difficulties in obtaining credible results and limiting their widespread use in children. Consequently, the noninvasive or least invasive diagnostic options are also gaining interest. Recently, saliva or capillary blood samples of allergen-specific immunoglobulin E (sIgE) have been detected as a viable option that offers a child-friendly procedure that increases compliance and early diagnosis (143). These methods reduce the physical and psychological burden of children and enable them to repeat the testing to monitor the disease progression or treatment impact.

Molecular allergen diagnostics has been particularly helpful in pediatric AR, where the actual sensitizing allergens can be determined at the molecular level. Proper and timely detection of the specific allergens substances, that contain HDM allergens Der p 1 and Der p 23, can result in specific management interventions and avoid unnecessary allergen avoidance that can adversely affect the quality of life. Moreover, molecular diagnostics may provide prognostic information that identifies children at higher risk of developing asthma and implement early interventions that might possibly change the disease pathway (66). One of them is the sensitization to Der p 23, which has been associated with asthma control status, and thus, renders it clinically relevant in respiratory allergic diseases among children. CRD integration in clinical practice, therefore, is a significant step towards precision medicine in pediatric AR.

Other than serological and molecular approaches, it has been recognized that nasal cytology is a convenient and informative procedure of measuring the inflammatory phenotype of AR in children. Nasal secretion cytology is not quite challenging to perform, it is least invasive and well tolerated. It will allow assessing cellular profiles including eosinophils, neutrophils, and mast cells that reveal the presence of underlying inflammatory processes and allow endotyping AR. Recent studies that apply ML techniques have applied nasal cytology data to classify pediatric AR into defined endotypes, which may be utilized to tailor treatment strategies (144). For example, specific anti-inflammatory agents can be used to treat children with eosinophil- and mast cell-dominated profiles. In addition, nasal cytology can be used to monitor disease activity and response to treatment over time that provides a dynamic assessment of the nasal mucosal environment.

Another complication is the LAR diagnosis in children. LAR is a type of localized inflammation of the nose without systemic atopy resulting in negative SPT and serum sIgE tests. Although LAR is well described in adults, it has not been well diagnosed in children due to the minimal awareness and the inability to diagnose it. The LAR diagnosis gold standard is NAPT that is technically difficult and is not readily available. Epidemiological data show that the geographical and methodological disparities in prevalence of LAR among children are extremely high, which explains the need of standard diagnostic guidelines and increased awareness of the clinical practice (145, 146). The clinical significance of LAR identification is that it can require various methodologies of control in contrast to classical AR.

Pediatric diagnosis and treatment of the AR is also complicated by the interaction of AR with comorbidities, such as asthma and otitis media. It is revealed that children with AR are likely to experience turbinate hypertrophy and nasal obstruction, which correlates with the severity of the disease and quality of life (147). Moreover, AR is associated with an increased prevalence of recurrent anterior epistaxis and dental-supporting tissue diseases, particularly among younger children and boys, which suggests the systemic impact of allergic inflammation (148, 149). These comorbidities must be clinically evaluated and dealt with in a multidisciplinary manner to give the best results.

Noncoding RNAs, microRNAs (miRNAs), long noncoding RNAs (lncRNAs), and circular RNAs (circRNAs) are the emerging molecular biomarkers that may help elucidate the molecular pathophysiology of pediatric AR and can be exploited as future diagnostic and therapeutic targets (150, 151). One such example is miR-4497, which has been reported to be down-regulated in allergic diseases in children and mediate type 2 inflammation and can be used as a noninvasive biomarker (151). Such molecular biomarkers in combination with clinical and cytological data can result in an improved diagnostic accuracy and enable individual medicine approaches.

In summary, low compliance with traditional invasive tests and disease phenotype variability are barriers to the diagnosis of AR in children. LAR diagnosis and the importance of new molecular biomarkers are also elements of the diagnostic image. A combination of all these innovations will assist in diagnosing early and accurately, prescribing individual treatment interventions and prognosis, which will eventually lead to an improvement in the management and quality of life of children with AR.

### 6.2 Deepening the diagnosis of LAR and AR comorbidities

LAR is now an autonomous clinical disease with nasal allergic symptoms without systemic atopy as evidenced by negative SPT and serum allergen-specific IgE (sIgE) levels. NAR diagnosis is made on the basis of NPT and/or nasal secretions sIgE which is a direct test of localized nasal mucosal reactivity. This diagnostic technique has clarified the epidemiology and clinical manifestation of LAR and transformed it into a disease phenomenon on its own, rather than a form of NAR or systemic AR (152–154).

Epidemiological studies indicate that LAR occurs in a large proportion of patients who had previously been diagnosed with NAR and that the prevalence rates of LAR vary greatly across regions and populations with higher prevalence reported in the western countries as compared to Asia (155, 156). LAR is a clinically equivalent of AR, it is featured by nasal obstruction, rhinorrhea, sneezing, and nasal itch and is often accompanied by eosinophilic inflammation and local IgE production in the nasal mucosa. It is important to note that comorbidities of asthma, atopic dermatitis, and conjunctivitis are also present in LAR patients, and this effect suggests the systemic effect of local nasal allergic reactions (154, 157).

The reference standard for LAR diagnosis, which induces local allergy and supports that the allergy is nasal-specific IgE-mediated hypersensitivity, is nasal allergy challenge (nasal allergen challenge NAC). However, NAC is resource-consuming and not universal, which contributes to the investigation of alternative diagnostic tools, including the basophil activation test (BAT) and nasal sIgE level by using mucosal brushings. These methods demonstrate promising sensitivity and specificity in distinguishing clinical and incidental local sensitization and systemic sensitization (153, 158). These diagnostic modalities combined help to make specific phenotyping of patients with rhinitis and differentiate between classical AR, LAR, dual AR (systemic and local sensitization coexisting) and NAR, which is necessary in the course of making specific therapeutic decisions.

The patients with AR with comorbidities, such as asthma, atopic dermatitis, or food allergy, need a systematic and comprehensive diagnostic workflow and optimal management. The workflow consists of the evaluation of upper and lower airway involvement, SPT, serum sIgE tests, and food challenge testing in case of need to assist the clinician to clarify the complex interplay between allergic sensitizations and clinical manifestations (159, 160). Molecular allergy diagnostics has taken on a larger role in this respect, offering component-resolved diagnostics, explaining patterns of cross-reactivity and profiles of polysensitization, and informing individualized AIT regimens and improving clinical outcomes (6, 161).

It is particularly noteworthy that AR and asthma are comorbidities, as these two conditions share pathophysiological mechanisms and tend to exacerbate each other, which leads to ineffective disease management and low quality of life. The quality of asthma control, pulmonary function, and quality of life associated with asthma are linked to the severity of the AR, and the two diseases have to be evaluated and treated together (160, 162, 163). In this respect, intranasal corticosteroids and antihistamines do not just enhance the nasal symptoms, but can be used to control asthma better. One of the issues, however, is medication adherence, especially in patients with mild symptoms and those who are afraid of adverse effects, and the education of patients and assistance of healthcare providers is important in this case (164, 165).

AR is often comorbid with such other disorders as chronic rhinosinusitis, nasal polyposis, and allergic conjunctivitis, which can complicate the clinical picture and involve multidisciplinary treatment (166, 167). Allergic disorders comorbidity also dictates the form of therapeutic choices and efficacy, including AIT and novel biologic interventions, which have demonstrated to be effective in numerous atopic diseases (168). Early identification of LAR and comorbidities in pediatric populations is vital since AR could be included in the so-called atopic march, and early AR could be a risk factor of additional asthma and food allergies (169, 170). The longitudinal studies also stress the necessity to monitor the trends of allergic sensitization and comorbidities over time and take the appropriate intervention (171).

In conclusion, the establishment of diagnostic ability of LAR by NPT and nasal sIgE detection have helped refine the comprehension of LAR as a distinct entity that has distinct epidemiological and clinical characteristics.The proposed change in the paradigm of the traditional systemic allergy testing to precise evaluation and integrated care is likely to provide improved patient outcomes in AR and comorbidities.

## 7 Limitations and challenges in precision diagnostics

While precision diagnostic technologies have significantly expanded the toolbox for AR assessment, several limitations and barriers remain that constrain their clinical adoption.

### 7.1 Cost and accessibility

Advanced molecular assays, CRD, multiplex platforms, and AI-based tools are associated with higher costs compared to conventional tests such as SPT and sIgE. This limits their accessibility, particularly in resource-limited settings, and may restrict widespread implementation in routine clinical practice.

### 7.2 Regulatory and standardization challenges

Many emerging technologies, including multiplex assays and AI algorithms, lack standardized protocols and regulatory approval across regions. Variability in assay design, data formats, and interpretation criteria hinders reproducibility and comparability between laboratories.

### 7.3 Clinical adoption barriers

Integration of advanced diagnostics into routine care is challenged by limited clinician familiarity, requirement for specialized interpretation, and the need for evidence demonstrating clear impact on patient management. AI tools, in particular, require robust validation and may be affected by biases in training datasets, incomplete or low-quality data, and limited generalizability.

### 7.4 Ethical and practical considerations

The use of AI and high-dimensional data raises ethical concerns regarding patient privacy, data security, and informed consent. Furthermore, complex molecular evaluation may generate incidental findings or overdiagnosis, which must be carefully managed in clinical decision-making.

In summary, while these technologies hold promise for precision medicine, their implementation must be carefully evaluated in the context of cost, accessibility, regulatory approval, standardization, and real-world clinical applicability.

## 8 Future perspectives in AR diagnosis

**Figure 4:**
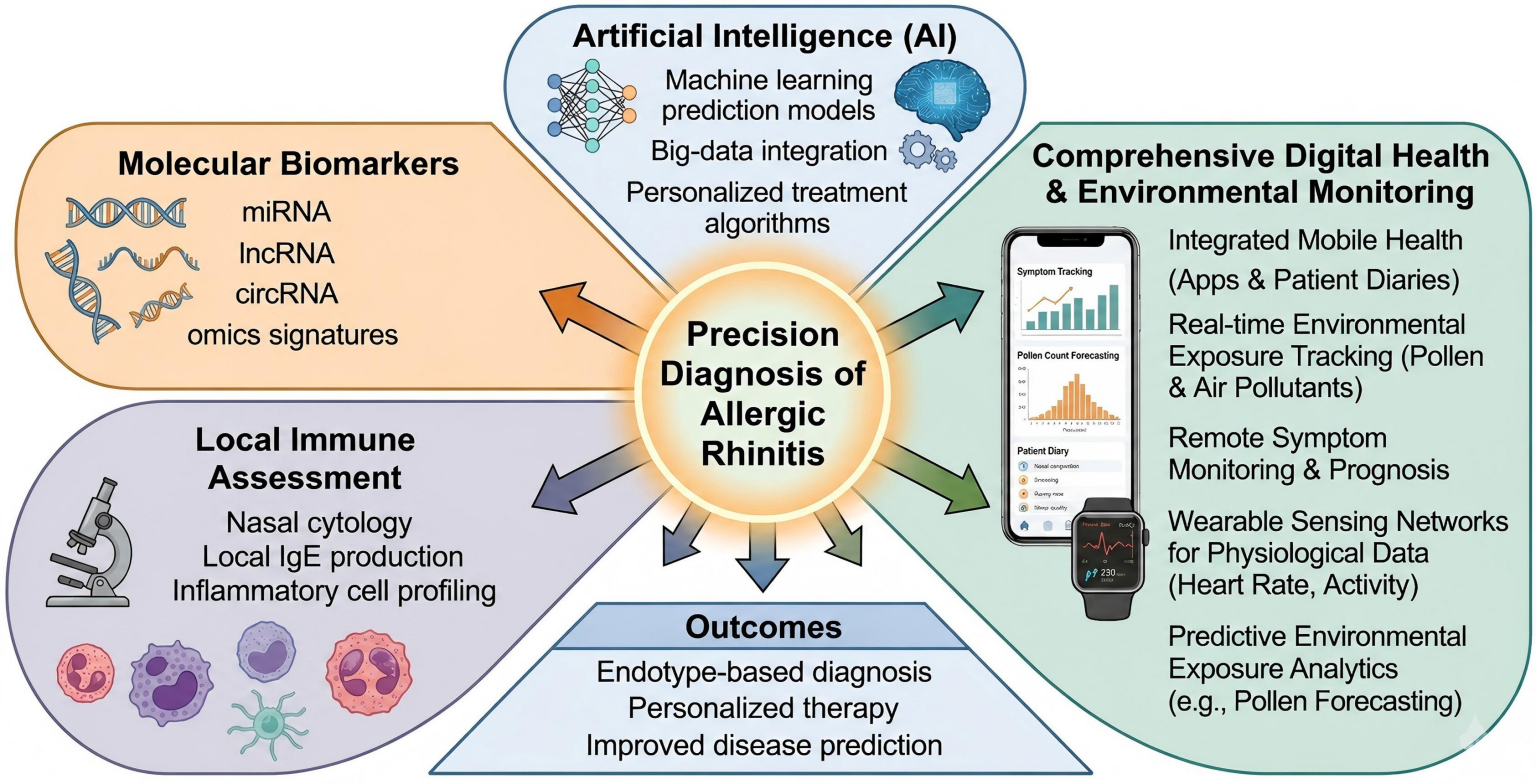
Future precision diagnostic framework for AR.

In recent years, advances in molecular biology and computational technologies have greatly expanded the tools available for AR research. Increasing evidence suggests that integrating molecular biomarkers with clinical and environmental data may improve the accuracy of disease classification and diagnosis. Recent advances in molecular biology, digital health technologies, and AI are transforming the diagnostic landscape of AR. The integration of multi-omics biomarkers, local immune profiling, and continuous symptom monitoring may enable a precision diagnostic framework that supports individualized disease management. Such multidimensional approaches are expected to play a central role in the future of AR diagnosis (Figure 4). As the field moves toward precision medicine for allergic rhinitis, it is important to distinguish between approaches that are potentially suitable for future guideline incorporation and those that require further investigation. Conventional assessments, including skin prick testing and serum-specific IgE, remain foundational. CRD may be applied for polysensitized patients, particularly when guiding immunotherapy selection. Functional and biomarker-based tests, such as nasal provocation testing, nasal biomarkers, and fractional exhaled nitric oxide (FeNO), can be considered in selected patient groups, including children and patients with LAR. On the other hand, several emerging technologies require further research before they can be recommended in guidelines. AI-based diagnostic models need multi-center validation, assessment of potential biases, evaluation of dataset size and quality, and integration into clinical workflows. Multi-omics approaches still require evidence regarding clinical utility, cost-effectiveness, and standardization. Imaging techniques need standardized protocols and demonstration of impact on patient outcomes. Clarifying these distinctions can help guide both future research priorities and the eventual translation of precision diagnostic tools into clinical practice.

Overall, while emerging diagnostic technologies have significantly expanded the toolbox for AR assessment, their clinical value should be interpreted within a broader context of diagnostic accuracy, cost-effectiveness, and real-world applicability. No single modality provides a definitive diagnosis in all cases, and over-reliance on highly sensitive molecular techniques may increase the risk of overdiagnosis and unnecessary interventions. Future diagnostic strategies should prioritize integrated approaches that combine clinical history, conventional testing, and advanced tools in a stepwise and cost-conscious manner, ensuring both diagnostic precision and practical feasibility.

## 9 Conclusion

The evolution of clinical diagnostic methods for AR reflects a progression of the classical *in vitro* and *in vivo* tests to the complex, multidimensional, and precision-focused diagnostic system. This change is important in the dynamic interaction between the development of scientific knowledge and its clinical implementation and the need to incorporate various approaches to promote the accuracy of diagnosis and inform therapeutic decision-making.

The introduction of molecular allergen diagnostics is one of the improvements in the clinical perspective. These techniques are superior to the conventional testing methods because they are able to subdivide the immunological basis of sensitization at the molecular level and give unparalleled specificity. This precision improves the diagnostic clarity and directly gives the option and customization of allergen-specific immunotherapy, thereby maximizing patient outcomes. However, it is necessary to balance the enthusiasm which comes with the use of molecular diagnostics and an awareness of their current constraints like accessibility, cost and the need to have standard interpretation systems among different populations.

Besides molecular diagnostics, local inflammatory biomarker testing and standardized NPT has proven valuable particularly in non-classical presentations such as LAR. The modalities provide critical data on local immune reactions that may not be discerned by systemic measurements, address diagnostic gaps, and stratify patients. The issue is to reconcile these approaches to the daily clinical practice and make them more reproducible and less cumbersome to the patients.

Objective evaluation of nasal functionality using sophisticated imaging and functional testing also enriches the diagnostic environment. The quantitative characterization of the pathophysiological changes of symptomatology assists clinicians to abandon the subjective symptom description and allows one to accurately monitor the progression of the disease and the effectiveness of treatment. In order to maximize the utilization of these technologies, however, one has to be cautious of the cost-effectiveness, training requirements, and clinical applicability of the technology.

Multi-omics strategies and AI represent the next stage in the development of AR diagnostics. These technologies, though still in early stages, in the largely translational research phase, may transform clinical practice by converting complex, multi-layered biological information into practical clinical information. The AI-based models will be able to identify new biomarkers, predict the disease progression, and even inform treatment decisions, representing an application of precision medicine principles. The challenges of data standardization, transparency of the algorithms and ethical issues of patient privacy are the key to the fulfillment of this potential.

Another issue that must be addressed is to ensure that these diagnostic modalities are seamlessly incorporated into single clinical pathways in the future. In order to have clinically stratified care, it will be needed to come up with stratified and cost effective diagnostic algorithms which will capitalize on each of the approaches. The emphasis on endotype-based diagnosis aligns with the overall trend of precision medicine and optimization of prevention, treatment, and management of AR. Such a important development necessitates continuous learning and adaptability of clinicians who must choose selectively among the available and new technologies to meet the heterogeneous needs of the patients in terms of diagnosis.

In conclusion, the AR diagnostics trajectory reflects the integration of technological innovation and clinical expertise. The future of patient-centered care will be defined by a trade-off between the potential of the most recent and most sophisticated molecular, functional, and computational tools and their feasibility. Interdisciplinary collaboration and the application of a holistic diagnostic philosophy can help the medical community to foster the accuracy, effectiveness, and optimization of AR management strategies, which, in its turn, will enhance patient outcomes and quality of life.
